# Simulating the whole brain as an alternative way to achieve AGI

**DOI:** 10.1002/qub2.6

**Published:** 2023-10-30

**Authors:** Jianfeng Feng

**Affiliations:** ^1^ Institute of Science and Technology for Brain‐Inspired Intelligence Fudan University Shanghai China

We have all seen the current attention, and even hype, on big models and artificial general intelligence (AGI). Indeed, many colleagues are arguing that we have achieved AGI with the current version of ChatGPT. Really? Looking back, it is not surprising to have a machine, even a normal calculator, which can outperform us. For example, a calculator can easily beat most of us on the multiplication of two large numbers with its speed, while a basic laptop can store many books, but humans have far weaker means of recall. So it is something that we have already got used to, that man‐made machines can perform *certain* tasks far better than us.

This general issue leads us back to the big original question: how to define intelligence? People have come up with many different definitions which could serve this purpose. For example, Stephen Hawking said “intelligence is the ability to adapt to change.” Along a similar line one of my close colleagues, the most cited psychologist, Trevor Robbins, who also won the Brain prize, has defined intelligence as “flexibility.” Others might argue that complex language might be the carrier of “intelligence,” which we, as human beings, own uniquely. I am more in favor of the latter: a chameleon can adapt to its environment, but does it have intelligence? Well it exhibits adaptation, a stimuli‐reaction circuit, but not in a creative or intelligent way. I recall that a recent publication in *Nature Communications* which claimed that the algorithm implemented by *Caenorhabditis elegans* can drive an automatic car [1]. If you download the paper and read it, you will realize that this is an over claim. Another, more mechanical way, to define/test intelligence is the Turing test, which has its pros and cons and has been both criticized and applied widely (Figure 1A).

**FIGURE 1:**
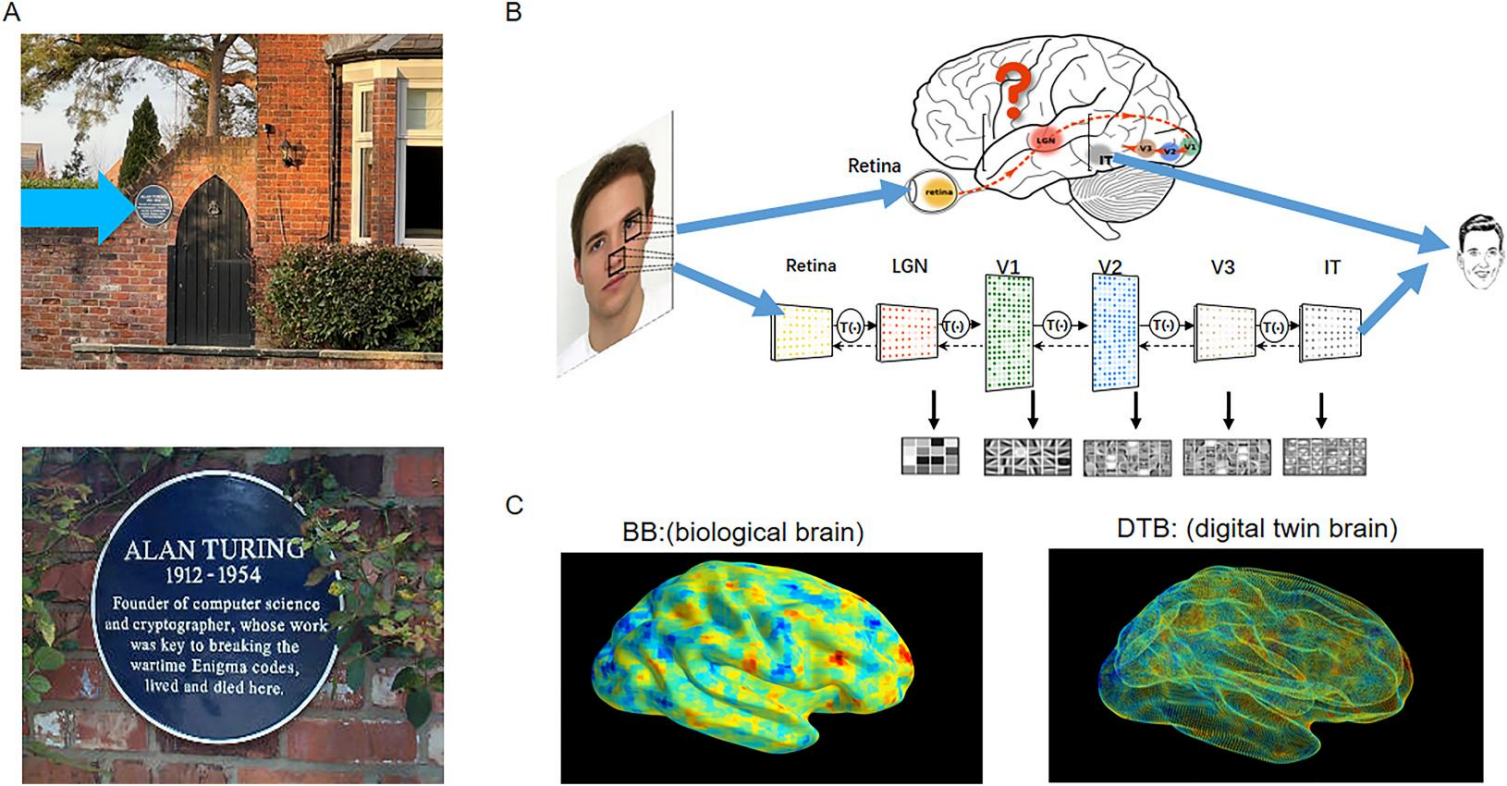
Can simulating the whole human brain be the only way to achieve AGI? (A) Turing’s home in a Manchester suburb area. The house was on sale for 1 M pounds in 2022. Top panel, the house. The arrow points to the plaque shown below. Bottom panel, the plaque. (B) Current deep neural network is a version of the ventral pathway, as indicated by arrows. The blank part, including emotion, for example, has largely not been included in the current AI approach. (C) Left panel, the biological brain. Right panel, the DTB. The correlation coefficient of the BOLD activity of the whole brain, of 20,000+ voxels, between the biological brain and DTB is above 90%. AGI, artificial general intelligence; DTB, digital twin brain.

Hence we have no criterion to really assess intelligence, at least, one which I am happy with. We can make a direct comparison between the human brain, the one which we all agree has the intelligence, and ChatGPT. First let us have a look of what ChatGPT has achieved. It can (amazingly) generate comprehensive answers, some of which are completely wrong! It can, sometimes, outperform an individual human. As we all know, the mechanism for ChatGPT, or all current machine learning, is to establish a correlation between different objects, and with ChatGPT the object is the word. With such a large model, with more than 100B parameters, a reasonable argument is that it obeys the Hegel’s law [2]: quantitative changes give rise to qualitative differences. It might be a good idea to carry out a similar analysis in, for example, the Ising model: when the size increases in ChatGPT, the correlation coefficient between nodes in the model diverges: a typical phase transition, which confirms the Hegel’s law.

Now let us look at our brain. Massachusetts Institute of Technology (MIT) neurobiologist Robert Desimone once wrote that ChatGPT only tries to mimic a very small portion of human brain, namely Broca’s area, where our language is generated. There is also another posterior brain region, called Wernicke’s region, where, in contrast to Broca’s area, comprehension of written and spoken language is performed. To fully understand the language in our brain is still a complex and active research activity, that is very much ongoing. Indeed, Jack Gallant laboratory, once reported that the semantic map is actually distributed over all of the human brain [3], which might simply imply that to fully mimic our language system, that is, to truly understand/simulate the semantic map rather than a correlation mapping, as in ChatGPT, we have to consider/simulate the whole human brain. As Richard Feynman famously said, “What I cannot create, I do not understand.”

Starting 10 years ago, the European Union (EU) launched its brain project: The Human Brain Project (HBP). This year is its final year of funding. The HBP intended to simulate the whole human brain, after its successful initial phase of the Blue Brain Project. At its initial phase, it quickly fell apart for various reasons. Currently one of the most successful outcomes of the HBP is The Virtual Brain (TVB) project led by our colleague Viktor Jirsa [4], who I worked with 17 years ago. In the TVB, a mean field model was developed to simulate the whole human brain activity: a set of equations is used to simulate each brain region. In this project, the participants went on to run a clinical trial in many hospitals in France and some very positive results arose, for the treatment of epilepsy [4]. The eBrain, an EU organization with Viktor Jirsa as its chief scientist, is the successor of the HBP. As an alternative to this, I note that a project to simulate the whole human brain was launched at Fudan University, with substantial support from the Shanghai municipal government in December 2018. We started this project with 86 billion neurons and around 100 trillion connection parameters, namely the conductance of AMPA, NMDA, GABA_A_ and GABA_B_, which were estimated. The model is called the Digital Twin Brain (DTB) [5, 6, 7] and is, without a doubt, a large model (Figure 1C). Last month, we successfully carried out the third run of the whole brain simulation using 13,000+ GPU cards.

What is the key difference between our brain and current computers (all these man‐made machines), from a mechanistic point of view? A simple answer is that our brain is a *probabilistic machine*: it calculates in a noisy or dynamic background. Many years ago, I had had a conversation with Wilfrid Rall, one of the pioneers of modeling single neurons in the National Institutes of Health (NIH). We spoke next to a small stream in an evening. I still vividly remember that he pointed to the river next to us and said “look at it, it is all spatiotemporal dynamics there, and I believe our brain behaves in the same way; an incoming signal is like a stick stuck into it, and it acts within a dynamic background.” To fully understand the dynamic operation of our brain, we should go beyond naïve static ways to analyze its dynamics, such as functional connectivity [8].

Why should we simulate the whole brain with 86 billion neurons simultaneously, a challenging task in computer science [5], in mathematics [6], and in biology [8]? We can certainly be in an easier position to simulate a brain region. We always argued that the current deep neural network simulates one visual pathway: the ventral pathway which terminates in the inferior temporal cortex and recognizes patterns, for example, our face. Figure 1B shows the ventral pathway and there is a huge blank area which we have not taken into account for the current artificial intelligence (AI) model. Our brain only works as a whole to function normally. In biological terms, the current AI approach has achieved with an in vitro experiment setup (to perform experiments on slice), but only an in vivo experiment will unravel the truth (to carry out the experiment with the intact brain), see Figure 1B. I am confident that to simulate the whole human brain at the cellular level provides us with the key to understand the complex brain spatiotemporal dynamics (cognitions) and subsequently achieve AGI.

By saying all of these, I am not arguing that the current progresses in AI research is not exciting and encouraging. At Fudan University, we are very much involved ourselves. Due to the current wave of AI research, our group benefits considerably: publication citations are higher than ever before and increase exponentially. The funding is steady and attracts not only more government money but also considerable amounts from industry. I have been asked by many funding agencies and organizations how to further facilitate current AI research. My answer is actually simple and practical: adding *IEEE Transactions on Pattern Analysis and Machine Intelligence (TPAMI)*, one of the top journals in AI research, to the promotion criterion for many universities, in addition to *Cell, Nature,* and *Science*.

## AUTHOR CONTRIBUTIONS

Jianfeng Feng drafted, revised and approved the final manuscript.

## CONFLICT OF INTEREST STATEMENT

Jianfeng Feng is one of Editorial Board Members of *Quantitative Biology*. He was excluded from the peer‐review process and all editorial decisions related to the acceptance and publication of this article. Peer‐review was handled independently by the other editors to minimize bias.

## ETHICS STATEMENT

This manuscript does not involve a research protocol requiring approval by the relevant institutional review board or ethics committee.
